# Efficacy of *Samsarjanakrama* in a patient with *Agnimandya* due to *vyadhi sankar*: A case study

**DOI:** 10.1016/j.jaim.2021.01.004

**Published:** 2021-02-27

**Authors:** Mukta, Mangalagowri V. Rao, Jyoti Arora

**Affiliations:** a Department of Swasthavritta, All India Institute of Ayurveda, New Delhi, 110076, India; b Department of Swasthavritta, Institute of Medical Sciences, Banaras Hindu University, Varanasi, India

**Keywords:** *Agnimandya*, *Vyadhi Sankara*, *Samsarjana krama*, *Agni*, *Ama*

## Abstract

Most of the people experience digestive problems like constipation, diarrhoea, acid eructations, loss of appetite etc. at some stage of life. These are the acute conditions that appear due to *Agnimandya* (reduced power of digestion, assimilation and metabolism). Conditions that may cause *Agnimandya* includes changes in diet and lifestyle and chronic diseased conditions. When the *Agnimandya* or the conditions that may cause *Agnimandya* persist for longer duration it turns into a life threatening disease. When *Doshas* (body humours) get aggravated, they affect the *Agni* (Power of digestion, assimilation and metabolism) and thus the food taken is not digested properly forming the *Ama* (morbid material). *Ama* when formed is accumulated in the body over the period of time, forming roots of many diseases.

In the present case study the patient has *Agnimandya*, because of the chronic metabolic conditions. The *Agnimandya* and consequent nutritional deficiencies are addressed, while preparing the treatment protocol. The central focus of *Ayurvedic* treatment is the management of *Agni* and the *Ama*. The diet was advised on the line of *Samsarjana Krama* (Sequential administration of liquid diet to normal diet to kindle the *Agni* or digestive power) and the effect of treatment was analysed after 1 month. The symptoms of the diseases were reduced along with the improvement in the *Agni* and the nutritional status.

## 1 Introduction

Impairment of the *Agni* is one of the most important etiological factor for causation of diseases as per *Ayurveda.* There are 4 types of *Agni – Samagani* (normal digestive, assimilation and metabolic power)*, Mandagni* (reduced power of digestiion, assimilation and metabolism) *Teekshnagni* (Intense power of digestion, assimilation and metabolism) and *Vishamagni* (Some time intense and some times reduced power of digestion, assimilation and metabolism) [1, Grahani chikitsa, ch 15/50-51] among them only *Samagni* is normal and other types being harmful. Impaired state of Agni results in various diseases or disease combinations. Most of the people experience digestive problems like constipation, diarrhoea, acid eructations, loss of appetite etc. at some stage of life. These are the acute conditions that appear due to *Agnimandya* (reduced power of digestion, assimilation and metabolism). Conditions that may cause *Agnimandya* include changes in diet and lifestyle and chronic diseased conditions. When the *Agnimandya* or the conditions that may cause *Agnimandya* persist for longer duration, it turns into a life threatening disease.

Aggravated *Kapha* is one among the important causes for *Agnimandya* (reduced power of digestion, assimilation and metabolism). leading to improper digestion of ingested food consequently producing *Ama*. Further, accumulated *Ama* in the due course of time, gives rise to various diseases. In the present case, the metabolic syndrome including components like obesity, hypertension, dyslipidemia, diabetes mellitus, CVD, are produced due to *Agnimandya,* and *Ama*.

In Ayurveda *“Vyadhi sankara”* is the term used for the above mentioned co-existence phenomenon in an individual [1, Nidana Sthana; Apasmara Nidana, ch 8/22]. And the reason for this co-existence according to *Ayurveda* is the similarity in the etiological factors, *Dosha* (body humours)*, Dushya* (body tissue) and *Srotas* (channels) involved in each of these diseases. As per Ayurveda the *Agnimandya* results in *Ama* leading to *Srotodushti* (derangement in channels of circulation) in the form of *Sanga* (obstruction) and *Vimarga gamana* (movement in abnormal direction) giving rise to diseases with distinct features pertaining to the deranged *Srotas (*channel*)*. Protection of *Agni* is the first and foremost principle of the *Ayurvedic* management [2, Chikitsa Sthana; Grahni Chikitsa, Ch 15/ 39-40]. *Agni* can be preserved by following correct rules for lifestyle and diet. *Ayurvedic* dietetics involves eight rules known as *“Ashta Ahara Vidhivisheshayatana”* [2, Vimana Sthana; Rasa Vimaniya, Ch 1/ 21]. These rules help in protection of *Agni* and maintenance of optimum nutritional status [2, Chikitsa Sthana; Grahni Chikitsa, Ch 15/ 39-40].

In the present study the patient is suffering from *Agnimandya* due to coexistence of various metabolic disorders, hence the line of treatment for *Agni dipana* is based on the *Samsarjana Krama* (Sequential administration of liquid diet to normal diet to kindle the *Agni* or digestive power) [2, Siddhi sthana; Kalpana Siddhi Ch 1/11-12]. The gradual inclusion of progressively heavy food from light food like *Manda* (liquid part only)*, Peya* (includes both liquid and rice grains)*, Vilepi* (More rice grains with little amount of liquid)*, Krishara* (Semi solid preparation made of pulse and rice) *to Bhakta* (Rice) or *Rotika* (Indian pan cake), *Yusha* (Soup made of pulses) in a sequential order in *Samsarjana krama (*Graduated dietetic protocol), enhances Agni [2, Siddhi Sthana; Kalpana Siddhi, Ch 1/12] and brings about better acclimatization.

## 2 Case report

### 2.1 Chief compalints

*Anannabhilasha* (loss of appetite) –since 3 weeks; *Adhovata and Urdhvavata* (flatulence and belching)- since 3 weeks; *Daurbalya in limbs* (weakness in limbs)- since 3 weeks.

### 2.2 History of present illness

A 59 years old female, visited Lifestyle OPD of AIIA, with complaints of *Anannabhilasha* (Loss of Appetite) along with early satiety, *Amlapitta* (Hyper-acidity), *Adhovata* and *Urdhvavata* (Flatulence and Belching) and general weakness along with tachycardia. Patient also complained of constipation. Disturbed sleep along with restlessness at night was also reported. Detailed Examination (Table 1) was done following *Trividha* and *Dashvidha Pareeksha[2, Vimana Sthana; Rogabhishagjitiye Ch 8/94]*.

**Table 1:** *SAMPRAPTI GHATAKA* (Components of pathogenesis).

*Samprapti Ghataka* (Components of pathogenesis)	Involvement
*Dosha* (body humours):	*Vata- Samana, Vyana and Udana,*
	*Pitta- Pachaka, Sadhaka,*
	*Kapha- Avalambaka*
*Dushya* (Body tissue)	*Rasa, Rakta, Mamsa, Meda, Majja, Sukra, Kleda, Vasa, Lasika, Oja*
*Agni* (Power of digestion, assimilation and metabolism):	*Jatharagni, Dhatvagni*
*Agni Dushti (*Type of derangement of *Agni)*	*Vishamagni*
*Ama* (Type of morbid matter)	*Jathargni* and *Dhatvagnimandyajanya Ama*
*Srotas* (channels)	*All*
*Sroto dushti Prakara* (type of derangement of channel)	*Sanga* (obstruction)*, Atipravritti* (Excessive production)
*Udbhava Sthana* (Site of orgin of disease)	*Amashaya* (Digestive tract)
*Sanchara Sthana* (Sites of movement)	*Sarvasharira* (entire body)*, Sarvadhatu* (all body tissues)
*Vyakti sthana* (Site of manifestation of disease)	*Sarvasharira* (Entire body)
*Adhishthana* (main seat of manifestation)	*Sarvasharira* (Entire body)
*Svabhava* (Nature of disease)	*Chirakari* (Chronic)

### 2.3 History of past illness

Patient has a history of diabetes (Type2), hypertension, hypothyroidism and CHD. Patient has a history of angioplasty and cholecystectomy.

### 2.4 Medication history

She is on allopathic medicines for above said multiple ailments- Metformin-500mg BD, Ecosprin-75mg OD, Eltroxin-75 mg OD, Gabapentine-OD, LASix –OD.

### 2.5 Family history

She has a family history of diabetes, hypertension and CHD.

### 2.6 Personal history

*Prakriti* was assessed using the PPAT (by S Rastogi and Prakriti) was found to be *Vata- Kaphaj*. Dietary information before intervention is provided in Table 2.

### 2.7 Vitals

Temperature – 96.3 °F; BP- 90/50 mm Hg; Pulse – 128 pm; Respiratory rate – 13 pm; RBS -287; No history of Addiction (both tobacco and alcohol); No Food Allergies; Allergic to Ibrupofen.

## 3 Detailed history before treatment

### 3.1 *Agni*

There is a significant change in the Appetite of the patient in the past 3 months and the amount of food intake has reduced to <75% (previously used to eat – 3 chapati now 1 chapati, no rice intake and the amount of vegetable from 1 full bowl to less than half bowl). Agni was assessed based on the “*Abhyavaran Shakti*” and “*Jaran Shakti*”. *Abhyavaran shakti* was assessed by the decrease in the amount of intake of food. *Jaran Shakti* was assessed by the patient’s complaint of flatulence.

Also the patient experiences early satiety and sweating and heaviness while taking meal.

### 3.2 Anthropometric measures

Height – 150 cm; Weight – 45 kgs (weight loss in past 3 months–10 kgs); Mid arm circumference – 12 cm; Waist circumference −36 cms; Waist – Hip Ratio – 0.5.

### 3.3 Features of depletion of *Dosha* (body humours) and *Dhatu* (body tissue)

*Ruksha kesha (d*ry hair), *Ruksha tvak* (dry skin), *Alpacheshtasu Hridrava* (Palpitations on little efforts), *Arasajnata (t*astelessness), *Hridrava (p*alpitations), *Hrullasa* (nausea), *Oshta sputana* (cracking of lips), pallor, decreased waist to hip ratio, *Sandhi shithilata*(Weakness of joints), *Bhangura Danta* (brittle tooth), *Danta Shoola* (pain in dentures), *Mukha Shosha* (dryness of mouth), *Daurbalya* (fatigue), *Durmana* (irritable).

## 4 Intervention

The patient was advised to take *Sadhita Manda* (liquid part after cooking 1 part of rice in 14 parts of water followed by seasoning with ghee, ginger, long pepper, black pepper and rock salt), for 2 days until the normal hunger is regained. This was followed by *Peya* and *Yusha* for 1 week then *Vilepi* for 1 week.

Followed by *Yavagu* for 1 week and then the appetite of patient gradually improved and diet was shifted to normal. The adherence and tolerability was assessed by daily follow up of the patient (on call). (The details of the intervention is provided in Table 3)

**Table 2:** Dietary intake (24 h Recall) before intervention.

Timings	Dietary items taken	Quantity with calories
Early morning	Tea with toast	2 toasts, 1 cup with 50 mL toned milk without sugar (87 calories + 60 calories)
Breakfast	Bread/Roti, Sabzi (mostly potato)	2 pieces/1 piece, 60–70 g (170/100 + 40 calories)
Lunch	Roti-1, Sabzi –seasonal (1/4th small bowl)	1 piece (30 gms), 50–60 g (100 + 40 calories)
Mid Evening	Tea	1 cup with 50 mL toned milk without sugar (60 calories)
Diner/Supper	Roti-1, Sabzi (1/4th small bowl)	1 Piece (30 gms), 50–60 g (100 + 40 calories)

**Remarks:** * Mostly patient skipped Dinners.

*Total calories being consumed by patient is 697 calories. Based on the Robinson formula (1983), your ideal weight is- **67.5 kgs** Calories reqd.is- **1575**.

* Calorie calculations [3].

Along with this patient was also taking fruits, tea and milk.

Apple (salt and pepper added) 100 gms, 60 calories twice daily; Pomegranate 100 gms, 80 calories once daily; Tea 120 ml (1 cup with toned milk without sugar), 60 calories; Roasted makhana (50 gms) - 180 calories; Marie biscuits [2, Vimana Sthana; Rasa Vimaniya, Ch 1/ 21] - 50 calories; Vegetable soup - 64 calories

Patients total calorie intake was gradually increased to 1200 calories keeping in mind the amount of nourishment required and the *Agni* of the patient.

## 5 Results

Patient was treated from October 2018 to November 2018. Outcome on all the parameters showed significant improvement. The weight of the patient increased from Kg to 48 Kg in 36 days (Table 4) Tachycardia improved and pulse rate at the 36th day was within normal limits (86 BPM). Food intake was increased to 75% which was less than 75% in the beginning of the study (measurement was done based on the required calorie intake. In the beginning of the study patient complained of early satiety along with various Dhatu kshaya symptoms like *Ruksha kesha (*Dry hair), *Ruksha Tvak* (Dry Skin), *Hridrva* (Palpitations on little efforts), *Arasagyta* (Tastelessness), *Hrullas* (Nausea), *Tvak sputana* (Cracking of lips), pallor, *Sandhi shithilata* (Weakness of joints), *Bhangura danta* (Brittle tooth), *Danta Shoola* (Pain in dentures), *Mukha shosha* (Dryness of mouth), *Daurbalya* (Fatigue), *Durmana* (Irritation). In the end of the study there was Decrease in palpitations and nausea but no improvement was noticed in twak and Kesha. Cracking of lips and pallor also decreased significantly, there was no weakness of joints, tooth ache, dryness of mouth and fatigue were also relieved. Patients diet intake pattern was also recorded in the beginning, a few changes were made including walking after meals and the gap between sleep and meals was increased (Table 5). There was improvement in the patient’s overall condition post treatment on 37^th^ day.

**Table 3:** Intervention Details

Intervention	Ingredients	Method of preparation	Properties	Anupana	Duration and frequency	Remarks
Sadhita Manda (medicated gruel without grains)	Red Rice, Water, *Trikatu* (Powder of black pepper, long pepper and ginger in equal proportion), *Saindhava lavana* (Rock salt), *Ghrita* (cow’s ghee)	1 part (1/4 cup- 50g) rice cooked in 14 parts (3.5 cups- 700 ml) water seasoned with ghee, ginger, long pepper black pepper and rock salt	*Dipana* (Kindles digestive power) and *Pachana* (Digests accumulated *Ama*)	Warm water (*Ushna jala*)	for 2 days three times a day (120 ml, 120calories) + 1/2 tsp Ghee (22.5 calories)	
*PEYA* (gruel with little grains)	Red rice, Water, *Trikatu* (Powder of black pepper, long pepper and ginger in equal proportion), *Saindhava lavana (*Rock salt), *Ghrita* (cow’s ghee)	1part rice/ Green gram (1/4 cup- 50g) cooked in 14 parts water with *Trikatu* (Powder of black pepper, long pepper and ginger in equal proportion), *Saindhava lavana* (Rock salt). There are few rice grains the *Peya*	Light (Quickly Digestable), *Dhatu pushtikara* (nourishes the tissues), *Balakaraka* (strengthening)	Warm water (Ushna jala)	for 1 week three times a day (120 gms, 140 calories)+ 1/2 tsp Ghee (22.5 calories) + Yusha (100 Calories)	Patient was asked to mix *Peya* and *Yusha* together
*VILEPI* (Rice gruel with less amount of water)	Red rice, Water, *Trikatu* (Powder of black pepper, long pepper and ginger in equal proportion), *Saindhava lavana* (Rock salt)	1 part rice (1/4 cup- 50g) cooked in 4 parts (1 cup – 200 ml) water with *Trikatu* and *Saindhava*	*Dhatuvardhaka* (nourishes the tissues), *Hrudya* (good for heart)	Buttermilk spiced	for 1 week 2 times a day (150 gms, 170 calories) + 1/2 tsp ghee (22.5 calories) + *Yusha* (150 gms, 100 calories)	Patient was eating it with *Yusha* (150 gms, 100 calories)
*YAVAGU* (thick gruel)	Red rice, Water, *Trikatu* (Powder of black pepper, long pepper and ginger in equal proportion), *Saindhava lavana* (Rock salt), *Ghrita* (cow’s ghee)	1 part (1/2 cup- 100g) rice cooked in 6 parts water (3 cups-600 ml) with *Trikatu* and *Saindhava*.	*Balya* (provides strength), *Tarpani* (provides satiety), *Vata Nashini* (alleviates Vata)	buttermilk spiced (200 ml - 80 calories)	for 1 week 2 times a day (150 gms, 170 calories)+ 1/2 tsp ghee (22.5 calories) + buttermilk spiced (200 ml - 80 calories)	This was used with the above said preparations as advocated.
*YUSHA* (lentil soup)	Moong Dal (Green Gram), water, *Trikatu* (Powder of black pepper, long pepper and ginger in equal proportion), *Saindhava lavana* (Rock salt), *Ghrita* (cow’s ghee)	moong Dal (1/2 cup-100 g) cooked in 14 parts (7 cups- 700ml) water with *Trikatu* and *Saindhava*.	*Balya* (provides strength), *Kanthya* (good for throat), *Laghu paka* (easy to digest) and *Kaphahara* (reduces *Kapha*)	buttermilk spiced	for 1 week 2 times a day (150 gms, 170 calories) + 1/2 tsp ghee (22 calories) + buttermilk spiced (200 ml - 80 calories)	

∗Calories calculation was done by the standard method [4].∗ calories are calculated for the food article as a whole and not for the individual ingredients.∗a full cup measures 200 gms/ml [4].

**Table 4:** Pre and post analysis of parameters.

S. No.	Parameters	Before treatment (9/10/2018)	After treatment (16/11/2018)
1	Weight	45 kgs (weight loss in past 3 months–10 kgs)	48 kgs
2	Pulse rate	128 Per minute	86 Per minute
3	RBS	287 gm/dl	162 gm/dl
4	Food intake	<75%	75%
5	Early satiety	Present	Absent
6	Sweating & nausea	Present	Sweating decreased, nausea absent
7	Bowel	On and off constipation with flatulence	Mild relief in constipation, no flatulence
8	*Rasa Kshaya (*Depletion of body fluids*)*	*Ruksha kesha (*Dry hair), *Ruksha Tvak* (Dry Skin), *Hridrva* (Palpitations on little efforts), *Arasagyta* (Tastelessness), *Hrullas* (Nausea)	Decrease in palpitations and nausea No improvement noticed in twak and Kesha
*9*	*Rakta Kshaya* (Depletion of blood)	*Tvak sputana* (Cracking of lips), pallor	Decrease in pallor, cracking of lips
10	*Mamsa Kshaya* (Depletion of flesh)	*Kshaya (*Mid arm circumference – 12 cm)	Improved to moderate (13 cms)
11	*Meda Kshaya* (Depletion of fat)	*Sandhi shithilata* (Weakness of joints) Waist – Hip Ratio – 0.5	Decrease in weakness of joints
12	*Asthi Kshaya* (Depletion of bone)	*Bhangura danta* (Brittle tooth), *Danta Shoola* (Pain in dentures)	Relief in tooth ache
13	*Majja Kshaya* (Depletion of bone marrow)	*Mukha shosha* (Dryness of mouth)	No dryness of mouth
14	*Oja Kshaya* (Depletion of factor responsible for immunity)	*Daurbalya* (Fatigue), *Durmana* (Irritation)	No fatigue

**Table 5:** Diet intake pattern.

Activities	Practice before treatment	Current practice	Remarks
Time of food: Breakfast F-8-9 am Lunch −2 pm, Dinner- 8–9 pm	Same time	No change	No change
*Ushnam Ashniyat -* Do u take food warm? If yes, When? Breakfast, Lunch and Dinner	3/3	3/3	No change
*Atimatra -* How often in a week, do you over eat? (Discomfort after food frequency)	Never	Never	No change
*Heenmatra -* Do you intentionally eat less? If yes, how often in a week	Yes	No	Nausea cured
*Ajalpan, Ahasan, Tanmana Bhunjeet ??-*What are your activities during your food intake? Breakfast, Lunch and Dinner (Activities like watching TV/Reading newspapers/Talking with friends etc.)	None	None	No change
*Atidrutam/Ativilambitam*- What is your average duration of food intake? (In Minutes) Breakfast, Lunch and Dinner 10–15 min each	Same time	No change	No change
Do you take food on regular times regularly? If irregular, how often in a week?	Regular 3/3	Regular 3/3	No change
Do you eat other than your regular meal times? If yes, how often?	No	No	No change
Activity after meals			Started walking after meals as advised
Break fast	Sedentary	Active	
Lunch	Sedentary	Active	
Dinner	Sedentary	Active	
Gap between meals and sleep	1 h	2 h	Increased as advised

## 6 Discussion

In case of wholesome food intake, medicine is not required and medicines won’t do any good in case of unwholesome food intake [1, Vimana Sthana; Rogabhishagjitiye, Ch 8 / 94]. By taking wrong type of food different *Doshas* gets aggravated, dry, light etc. food items having similar properties as *Vata*, increases the *Vata* [2, Chikitsa Sthana; Grahni Chikitsa Ch 28/15] and heavy foods, oily foods increases the *Kapha* (Sutra 1/66, Page 18) [2, Sutra Sthana; Deerghanjivitiya, Ch 1/ 66]. When *Doshas* are aggravated they affect the *Agni* in different ways. Because of *Vata* sometimes there is *Mandagni* and sometimes *Teekshnagni* [5]. The root cause of all the diseases is *Agnimandya.* In this particular case the patient has a disease complex of hypothyroidism, diabetes mellitus, hypertension and cardiac disorders. The first problem that patient suffered was hypothyroidism, which is otherwise correlated to *Medo Dhatvagnimandya* [6], followed by other diseases. Diabetes mellitus is a type of *Vataja Prameha*, *Agnimandya* due to *Vata* [5] is thus one of the outcomes of *D.mellitus* [2, Nidana Sthana; Prameha Nidana, Ch 4 /48-49]. *Agnimandya* plays a very crucial role in the pathogenesis of almost all the above mentioned diseases in the patient. Hence, correction of *Agni* is the most important part in the treatment.

Apart from *Agnidipana* the other factors that should be considered, while prescribing a dietary intervention is the nutrient quality of the food articles. The *Manda, Peya* and *Vilepi* are taken in the current study, because these food items are time tested for enhancement of *Agni*, and are used in *Samsarjana Krama* also [2, Sidhi Sthana; Vamanvirechan vyapad Sidhi, Ch 6 /24]. *Manda, Peya and Vilepi* were prepared using rice and water (in varying amount), to gradually increase the *Agni. Manda* is easily digestible as it is iso-osmolar with the human body fluids (Osmolarity is the amount of total number of solute particles in a liter of liquid [7]). In *Peya* the amount of grains is more as compared to the *Manda*, but the amount of water was kept equal (14 parts), the food is also and brought about *Raktavriddhi* the fuel for *Agni*. Apart from enhancing the *Agni* these dietary items are high in nutrition and hence cover the nutrient loss also.

While giving the various preparations *‘Trikatu’ and ‘Ghrita’* were added as adjuvant, both *Trikatu* [5] and *Ghrita* act as *Deepana* (enhance digestion) and thus enhance the absorption of nutrients in food and active principles in medicines. *Trikatu* helps in digestion of *Ama* formed due to *Agnimandya* and *Trikatu* also prevents further *Ama* formation [8, ch 38/ 59]. *Ghrita* reduces the dryness due to its *Snigdhata* (unctuousness). As per eight factors of food intake (*Ashta Ahara Vidhi*) intake of unctuous food is advocated [2, Vimana Sthana; Rasa Vimaniya, Ch 1/21], in this case intake of ghee enhances *Agni. Peya and Vilepi* were advised to be taken with *Mudga Yusha,* as it breaks the monotony of the taste and also enhances the nutritive value by adding the limiting protein methionine in the rice meal. Warm water was advised after the meals as it quenches the thirst and also it helps in enhancement of the Agni and is always conducive to the human beings [8, ch 45/39-40]. Also, *Takra* (butter milk) was advised along with the *Vilepi,* as it enhances the *Agni* and also reduces obstruction in channel due to *lekhana* (scraping) property [8, ch 45/ 74].

Apart from the dietary preparations given at the meal time, patient was also taking fruits and tea. Only apple and Pomegranate were advised to be taken with the seasoning of salt and pepper. Apple is high in nutrients, with salt and pepper there is increased digestibility and absorption. Pomegranate helped to reduce palpitation due to *Pittahara, Hridya* (cardiac tonic) property and brought about *Raktavriddhi* [9]. by rich minerals, vitamins and anti-oxidants. As the patient was accustomed for intake of Tea, it was restricted to only 2 cups/day. Lifestyle of the patient before treatment was well managed and hence no changes were made.

## 7 Conclusion

The case mentioned above was diagnosed with *Vyadhi Sankara* in terms of *Ayurveda*, patient’s condition was deteriorating, because of the *Agnimandya*. The dietary advice was given on the line of *S. krama* to improve the status of *Agni,* which was the root cause for multiple ailments. The *Agni* improved and also nutritional status showed a little improvement with relief of symptoms like nausea, palpitation, pallor and cracking of lips after treatment.

## 8 Future recommendations

Cases of chronic *Agnimandya* can be treated in the line of *Samsarjanakrama* with success. This line of dietary intervention not only enhances the *Agni*, but also improves the nutritional status.

## 9 Strengths and limitations

There is very limited work on how Ayurvedic diet can help a patient in various diseases. And a very little published work is available on Ayurvedic diet as an intervention; the strength of this work is that the author has used only Ayurvedic methods of diet and lifestyle assessment and intervention.

Limitations – this work could have been more comprehensive, if the Ayurvedic assessment scales for nutritional status and quality of life were available.

## Source(s) of funding

None.

## Conflict of interest

None.
