# Supplementary material for: Efficacy of Samsarjanakrama in a patient with Agnimandya due to vyadhi sankar: A case study
Source: J Ayurveda Integr Med. 2021 Feb 27;12(1):182–6. doi: 10.1016/j.jaim.2021.01.004 (PMC8039330; doi:10.1016/j.jaim.2021.01.004)
Supplement: Multimedia component 2 [file mmc2.pdf]

REVISED VERSION OF PROTOTYPE PRAKRITI ANALYSIS TOOL (PPAT)

By Sanjeev Rastogi (Ref: Ayu. 2012 Apr-Jun; 33(2): 209–218.)

Features to identify Kapha components

(Each *Guna* corresponds to 96 points. Feature scores are divided as per the number of total features available to a particular *Guna*.)

No.	*Guna (Qualities)*	Clinical feature	Method of observation	Score	Yes/No
**1.**	*Snigdha (Unctuous)*	Oily skin	Inspection	96	**No**
**2.**	*Shlakshana (Smooth)*	Smooth skin	Palpation	96	**No**
**3.**	*Mridu (Soft)*	Fair complexion<br>Good looking face	Inspection<br>Inspection	48<br>48	**Yes**
**4.**	*Sara (Mobile)*	Compact and muscular body	Inspection and palpation	96	**No**
**5.**	*Sandra (Dense)*	Well-formed, proportionate body parts<br>Well-nourished body parts	Inspection<br>Inspection	48<br>48	**Yes**<br>**No**
**6.**	*Manda (Slow)*	Slow motor activity<br>Slow conversation<br>Slow eating	Interrogation<br>Interrogation<br>Interrogation	32<br>32<br>32	**No**<br>**No**<br>**No**
**7.**	*Staimitya (Stable)*	Delayed beginning of activities<br>Less anxious	Interrogation<br>Interrogation	48<br>48	**No**<br>**No**
**8.**	*Guru (Heavy)*	Slow and constant walking speed	Interrogation	96	**No**
**9.**	*Sheeta (Cold)*	Less appetite<br>Less thirst<br>Less sweating<br>Tolerant	Interrogation<br>Interrogation<br>Interrogation<br>Interrogation	24<br>24<br>24<br>24	**No**<br>**No**<br>**No**<br>**No**
**10.**	*Picchila (Slimy)*	Compact joints	Inspection	96	**No**

Total *kapha* score- 144/1056

Features to Identify Pitta component

No.	*Guna*	Clinical feature	Method of observation	Score	Yes/No
1.	*Ushna (Hot)*	Intolerant to heat<br>Soft textured hair and skin<br>Fair complexion<br>Increased presence of mole<br>Increased appetite and thirst<br>Premature graying and fall of hair	Interrogation<br>Inspection<br>Inspection<br>Inspection<br>Interrogation<br>Inspection	44<br>44<br>44<br>44<br>44<br>44	No<br>No<br>Yes<br>No<br>No<br>No
2.	*Tikshna (Piersing)*	Eat and drink good quantity of food at a time<br>Instantly reacting, argumentative<br>Intolerant to discomforts<br>Unable to tolerate hunger and thirst	Interrogation<br>Interrogation<br>Interrogation<br>Interrogation	66<br>66<br>66<br>66	No<br>No<br>No<br>No
3.	*Drava (Liquid)*	Lax and soft flesh and joints<br>Profuse sweat, urine, and stool formation	Inspection<br>Interrogation	132<br>132	No<br>No
4.	*Visra (Bad smelling)*	Increased and offensive odor from armpit, head, and body	Interrogation	264	No

Total *Pitta* score- 44/1056

Features to identify Vata components

(Each *guna* corresponds to 132 points. Feature scores are divided as per the number of total features available to a particular *guna*.)

No.	*Guna*	Clinical feature	Method of observation	Score	Yes/No
1.	*Ruksha(Dry)*	Dry skin<br>Poorly formed and nourished body<br>Interrupted and unpleasant voice<br>Interrupted, reduced, and shallow sleep	Inspection<br>Inspection<br>Hearing<br>Interrogation	33<br>33<br>33<br>33	Yes<br>Yes<br>No<br>Yes
2.	*Laghu (Light)*	Fast walking speed<br>Fast intake of food<br>Fast movements, hyperactive	Interrogation<br>Interrogation<br>Inspection, Interrogation	44<br>44<br>44	No<br>Yes<br>No
3.	*Chala (Mobile)*	Unstable joints and body parts (moves them while sitting)	Inspection, Interrogation	132	No
4.	*Bahu (Large)*	Increased number of visible tendons and veins on extremities<br>Over talkative	Inspection<br>Inspection, Interrogation	66<br>66	Yes<br>No
5.	*Shighra(Fast)*	Quick indulgence in some activity<br>Increased anxiety<br>Quick attachment and detachment<br>Fearfulness, timidness<br>Quick understanding and grasping<br>Less memory	Interrogation<br>Interrogation<br>Interrogation<br>Interrogation<br>Interrogation<br>Interrogation	22<br>22<br>22<br>22<br>22<br>22	Yes<br>No<br>Yes<br>No<br>Yes<br>No
6.	*Sheeta (Cold)*	Intolerant to cold (does not like)<br>Prone to cold-induced illness (common cold, URTI)	Interrogation<br>Interrogation	66<br>66	Yes<br>No
7.	*Parush (Rough)*	Rough hair, nail, skin, foot, and hand	Inspection	132	Yes
8.	*Vishada (Non Slimy)*	Prominent joints<br>Crepitus in joints while moving	Inspection<br>Interrogation	66<br>66	Yes<br>Yes

Total *Vata* score 605/1056

Total composite score is 1056 for each *Dosha*. Individual *Dosha* scores are the cumulative sum of scores obtained against the positive features as per the recorded responses.

Physical features like compact body and musculature are to be judged in accordance with the gender, ethnic, and geographic standards
